# Supplementary material for: Effects of structured small-group student talk as collaborative prewriting discussions on Chinese university EFL students’ individual writing: A quasi-experimental study
Source: PLoS One. 2021 May 28;16(5):e0251569. doi: 10.1371/journal.pone.0251569 (PMC8162705; doi:10.1371/journal.pone.0251569)
Supplement: S1 Appendix — (DOCX) [file pone.0251569.s001.docx]

**Appendix A**

Writing topic for tests:

Nowadays, our life is getting a lot simpler and more convenient because of various intelligent machines. However, some people think that our brains will get lazy in a world run by intelligent machines. Write a composition of at least 200 words on the following topic: *With intelligent machines to do the thinking, will our brains get lazy*?

Please follow these three steps to plan for your writing:

1. Giving your opinions on agreeing or disagreeing with the writing topic;
2. Generating and evaluating ideas and evidence that are for and against the writing topic;
3. Selecting and organizing ideas and evidence into a writing plan.
