# Supplementary material for: Effects of structured small-group student talk as collaborative prewriting discussions on Chinese university EFL students’ individual writing: A quasi-experimental study
Source: PLoS One. 2021 May 28;16(5):e0251569. doi: 10.1371/journal.pone.0251569 (PMC8162705; doi:10.1371/journal.pone.0251569)
Supplement: S2 Appendix — (DOCX) [file pone.0251569.s002.docx]

**Appendix B**

**Argumentative Writing Rubric**

(Jacobs et al., 1981)

| **ASPECT** | **SCORE** | **LEVEL/CRITERIA** |
| --- | --- | --- |
|  | 30-27  26-22  21-17  16-13 | EXCELLENT TO VERY GOOD: knowledgeable •substantive •thorough development of thesis • relevant to assigned topic  GOOD TO AVERAGE: some knowledge of subject •adequate range •limited development of thesis •mostly relevant to the topic, but lacks detail  FAIR TO POOR: limited knowledge of subject •little substance •inadequate development of topic  VERY POOR: does not show knowledge of subject •non-substantive •not pertinent •OR not enough to evaluate |
| 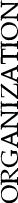 | 20-18  17-14  13-10  9-7 | EXCELLENT TO VERY GOOD: fluent expression •ideas clearly  stated/ supported •succinct •well-organized •logical sequencing • cohesive  GOOD TO AVERAGE: somewhat choppy • loosely organized but main  ideas stand out • limited support • logical but incomplete sequencing  FAIR TO POOR: non-fluent •ideas confused or disconnected •lacks  logical sequencing and development  VERY POOR: does not communicate • no organization • OR not enough to  evaluate |
| 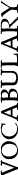 | 20-18  17-14  13-10  9-7 | EXCELLENT TO VERY GOOD: sophisticated range •effective word/ idiom choice and usage •word form mastery •appropriate register  GOOD TO AVERAGE: adequate range •occasional errors of word/ idiom form, choice, usage *but meaning not obscured*  FAIR TO POOR: limited range •frequent errors of word/ idiom form, choice, usage •*meaning confused or obscured*  VERY POOR: essential translation •little knowledge of English vocabulary, idioms, word form •OR not enough to evaluate |
| 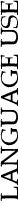 | 25-22  21-18  17-11    10-5 | EXCELLENT TO VERY GOOD: effective complex constructions • few errors of agreement, tense, number, word order/ function, articles, pronouns, prepositions  GOOD TO AVERAGE: effective but simple constructions •minor problems in complex constructions •several errors of agreement, tense, number, word order/ function, articles, pronouns, prepositions but *meaning seldom obscured*  FAIR TO POOR: major problems in simple/ complex constructions •frequent errors of negation, agreement, tense, number, word order/ function, articles, pronouns, prepositions and/ or fragments, run-ons, deletions •meaning confused or obscured  VERY POOR: virtually no mastery of sentence construction rules •dominated by errors •does not communicate • OR not enough to evaluate |
| 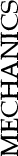 | 5  4  3  2 | EXCELLENT TO VERY GOOD: demonstrates mastery of conventions •few errors of spelling, punctuation, capitalization, paragraphing  GOOD TO AVERAGE: occasional errors of spelling, punctuation, capitalization, paragraphing but *meaning not obscured*  FAIR TO POOR: frequent errors of spelling, punctuation, capitalization, paragraphing •poor handwriting •meaning confused or obscured  VERY POOR: no mastery of conventions •dominated by errors of spelling, punctuation, capitalization, paragraphing •handwriting illegible •OR not enough to evaluate |
